# Response to brentuximab vedotin versus physician’s choice by CD30 expression and large cell transformation status in patients with mycosis fungoides: An ALCANZA sub-analysis

**DOI:** 10.1016/j.ejca.2021.01.054

**Published:** 2021-03-29

**Authors:** Youn H. Kim, H. Miles Prince, Sean Whittaker, Steven M. Horwitz, Madeleine Duvic, Oliver Bechter, Jose A. Sanches, Rudolf Stadler, Julia Scarisbrick, Pietro Quaglino, Pier Luigi Zinzani, Pascal Wolter, Herbert Eradat, Lauren C. Pinter-Brown, Pablo L. Ortiz-Romero, Oleg E. Akilov, Judith Trotman, Kerry Taylor, Michael Weichenthal, Jan Walewski, David Fisher, Marise McNeeley, Alejandro A. Gru, Lisa Brown, M. Corinna Palanca-Wessels, Julie Lisano, Matthew Onsum, Veronica Bunn, Meredith Little, William L. Trepicchio, Reinhard Dummer

**Affiliations:** a Dermatology and Medicine, Stanford University School of Medicine and Cancer Institute, 780 Welch Road, CJ220D, 94305, Stanford, CA, USA; b Department of Haematology, University of Melbourne, 140 Clarendon Street, 3002, East Melbourne, Australia; c St Johns Institute of Dermatology, Guys and St Thomas NHS Foundation Trust, St Thomas Street, SE1 7EL, London, UK; d Department of Medicine, Lymphoma Service, Memorial Sloan Kettering Cancer Center, 1275 York Avenue, 10065, New York, NY, USA; e Department of Dermatology, The University of Texas MD Anderson Cancer Center, 1515 Holcombe Blvd, Unit 1452, 77030, Houston, TX, USA; f Department of General Medical Oncology, Leuven Cancer Institute, University Hospitals Leuven, Herestraat 49, 3000, Leuven, Belgium; g Department of Dermatology, University of São Paulo Medical School, Av. Dr. Enéas de Carvalho Aguiar 255, S. 3068, 05403-000, São Paulo, Brazil; h University Clinic for Dermatology, Johannes Wesling Medical Centre, Hans-Nolte-Str. 1, D-32429, Minden, Germany; i Department of Dermatology, Cutaneous Lymphoma Service, University Hospital Birmingham, Mindelsohn Way, B15 2TH, Birmingham, UK; j Department of Medical Sciences, Dermatologic Clinic, University of Turin, Via Cherasco 23, 10126, Turin, Italy; k Institute of Hematology ‘Seràgnoli’, University of Bologna, Via Massarenti 9, 40138, Bologna, Italy; l Department of Internal Medicine/Medical Oncology, Klinik St. Josef, St Vith, Klosterstrasse 9, 4780, St Vith, Belgium; m Department of Hematology-Oncology, David Geffen School of Medicine at UCLA, 2020 Santa Monica Blvd Suite 600, 90404, Los Angeles, CA, USA; n Internal Medicine, Division of Hematology-Oncology, Chao Family Comprehensive Cancer Center, University of California, 101 The City Drive, 92868, Irvine, CA, USA; o Department of Dermatology, Hospital 12 de Octubre. Institute I+12. Medical School. University Complutense, Av Córdoba s/n, 28041, Madrid, Spain; p Department of Dermatology, University of Pittsburgh, 200 Lothrop Street, 15213, Pittsburgh, PA, USA; q Department of Hematology, Concord Repatriation General Hospital, University of Sydney, Hospital Road, 2139, Concord, Sydney, Australia; r Department of Hematology, ICON Cancer Care, 293 Vulture Street, 4101, South Brisbane, Australia; s Department of Dermatology, University Hospital of Schleswig-Holstein, Arnold Heller Str.3, 24105, Kiel, Germany; t Department of Lymphoid Malignancies, Maria Sklodowska-Curie National Research Institute of Oncology, 5 W. K. Roentgen, 02-781, Warsaw, Poland; u Department of Medical Oncology, Dana-Farber Cancer Institute, 450 Brookline Avenue, 02215, Boston, MA, USA; v Department of Anatomic Pathology for Clinical Trials, Quest Diagnostics, 1 Malcolm Avenue, 07608, Teterboro, NJ, USA; w Department of Pathology, University of Virginia, School of Medicine, 2730 Hunt Country Ln, 22901, Charlottesville, VA, USA; x Seagen Inc, 21823 30th Drive Southeast, 98021, Bothell, WA, USA; y Millennium Pharmaceuticals Inc., a wholly owned subsidiary of Takeda Pharmaceutical Company Ltd, 40 Landsdowne Street, 02139, Cambridge, MA, USA; z Department of Dermatology, Skin Cancer Center, University Hospital Zürich, Gloriastrasse 31, 8091, Zürich, Switzerland

**Keywords:** Antibody-drug conjugate, Brentuximab vedotin, CD30, Cutaneous T-cell lymphoma, Efficacy, Large-cell transformation, Mycosis fungoides, Objective response, Progression-free survival, Safety

## Abstract

**Introduction:**

Mycosis fungoides (MF), the most common type of cutaneous T-cell lymphoma, can lead to disfiguring lesions, debilitating pruritus and frequent skin infections. This study assessed response to brentuximab vedotin in patients with MF in the phase III ALCANZA study.

**Methods:**

Baseline CD30 levels and large-cell transformation (LCT) status were centrally reviewed in patients with previously-treated CD30-positive MF using ≥2 skin biopsies obtained at screening; eligible patients required ≥1 biopsy with ≥10% CD30 expression. Patients were categorised as CD30_min_ < 10% (≥1 biopsy with <10% CD30 expression), or CD30_min_ ≥ 10% (all biopsies with ≥10% CD30 expression) and baseline LCT present or absent. Efficacy analyses were the proportion of patients with objective response lasting ≥4 months (ORR4) and progression-free survival (PFS).

**Results:**

Clinical activity with brentuximab vedotin was observed across all CD30 expression levels in patients with ≥1 biopsy showing ≥10% CD30 expression. Superior ORR4 was observed with brentuximab vedotin versus physician’s choice in patients: with CD30_min_ < 10% (40.9% versus 9.5%), with CD30_min_ ≥ 10% (57.1% versus 10.3%), with LCT (64.7% versus 17.6%) and without LCT (38.7% versus 6.5%). Brentuximab vedotin improved median PFS versus physician’s choice in patients: with CD30_min_ < 10% (16.7 versus 2.3 months), with CD30_min_ ≥ 10% (15.5 versus 3.9 months), with LCT (15.5 versus 2.8 months) and without LCT (16.1 versus 3.5 months). Safety profiles were generally comparable across subgroups.

**Conclusion:**

These exploratory analyses demonstrated that brentuximab vedotin improved rates of ORR4 and PFS versus physician’s choice in patients with CD30-positive MF and ≥1 biopsy showing ≥10% CD30 expression, regardless of LCT status.

**Clinical trial registration:**

Clinicaltrials.gov, NCT01578499.

## 1. Introduction

Cutaneous T-cell lymphomas (CTCL) often have chronic courses and lead to disfiguring lesions, debilitating pruritus and frequent skin infections [1–3]. Mycosis fungoides (MF) is the most common CTCL subtype and patients frequently present with skin patches and/or plaques. Patients with advanced-stage MF may have skin tumours, erythroderma or extracutaneous disease [4]. Early-stage MF is primarily treated with skin-directed therapies, whereas in advanced-stage disease or refractory early-stage disease, systemic therapies are often used.

Diagnostic and clinical management of CTCL includes at least one skin biopsy assessed by expert dermatopathological evaluation, often utilising immunohistochemistry (IHC) staining. Both primary cutaneous anaplastic large cell lymphoma (pcALCL) and MF are characterised by expression of cell-surface CD30 antigen; pcALCL is characterised by a high level of CD30 expression (≥75% of tumour cells) [4], whereas MF may express CD30 to a more variable degree (<1–100%) [5–8]. Technical limitations of detecting low levels of CD30 expression for patients with MF, along with inter-patient, intra-patient and inter-lesional variability between the skin and lymph node of CD30 expression, have been reported [6,8,9].

Large cell transformation (LCT) of MF is defined as the presence of ≥25% of aberrant T-cells with large cell morphology (and/or large cell nodules) and is often associated with aggressive clinical course and inferior prognosis [10–12]. Some reports suggest the presence of LCT is associated with higher levels of CD30 expression in MF patients; however, CD30 expression is not required for the determination of LCT [13]. Further, the presence of LCT has been reported in >50% of patients diagnosed with advanced-stage (IIB–IV) MF [11,12].

The phase III ALCANZA trial (NCT01578499) evaluated the efficacy and safety of brentuximab vedotin versus physician’s choice (PC) of methotrexate or bexarotene in patients with previously-treated CD30-positive MF or pcALCL who required systemic therapy [14]. Selection of methotrexate or bexarotene in the PC arm was made by the treating physician based upon the patient’s diagnosis (MF or pcALCL), comorbidities, prior use of either agent, and availability of treatment at the participating centre. In patients with MF, CD30 expression was evaluated by IHC assessment of ≥2 skin biopsies from separate lesions. ALCANZA primary results demonstrated the superiority of brentuximab vedotin over PC, with significant improvements in all primary and key secondary endpoints, including objective response rate lasting ≥4 months (ORR4 [56.3% versus 12.5%; p < 0.0001]), complete remission rate (16% versus 2%; p = 0.0046), median progression-free survival ([PFS] 16.7 versus 3.5 months; hazard ratio [HR]: 0.270 [95% CI: 0.169–0.430]; p < 0.0001) and mean maximum reduction in Skindex-29 score (−27.96 [standard deviation (SD): 26.877] versus −8.62 [SD: 17.013]; p < 0.0001) [14].

This exploratory, *post hoc* analysis of patients with MF enrolled in ALCANZA evaluates whether baseline CD30 expression level impacted the efficacy and safety of brentuximab vedotin and retrospectively summarises the proportion and outcomes of patients with LCT at the time of enrollment.

## 2. Patients and methods

### 2.1. Patients

ALCANZA enrolled adult patients (aged ≥18 years) with CD30-positive MF (n = 100) or pcALCL (n = 31) who had received ≥1 previous systemic therapy (including radiotherapy for pcALCL), and had an Eastern Cooperative Oncology Group performance status of 0–2. This analysis was limited to patients with CD30-positive MF only.

For confirmation of eligibility, patients with MF were required to undergo ≥2 skin biopsies of patch, plaque or tumour lesions, selected at the investigator’s discretion, for central confirmation of CD30 expression by IHC. Each biopsy was ≥2 mm in diameter and obtained from separate skin lesions, where possible. Patients were eligible if they had at least one biopsy with ≥10% CD30-positive malignant cells or lymphoid infiltrate by central pathology review and were not limited to the number of total biopsies [14].

### 2.2. Study objectives

As reported previously [14], ALCANZA was an international, open-label, randomised, phase III, multi-centre study to assess the efficacy and safety of brentuximab vedotin compared with PC. Local ethics committees or institutional review boards approved the protocol, and all patients provided written informed consent.

The aims of these *post hoc* analyses were to determine the relationship between baseline CD30 expression and response to brentuximab vedotin and to summarise LCT status and outcomes.

### 2.3. Assessments

**Fig. 1.**
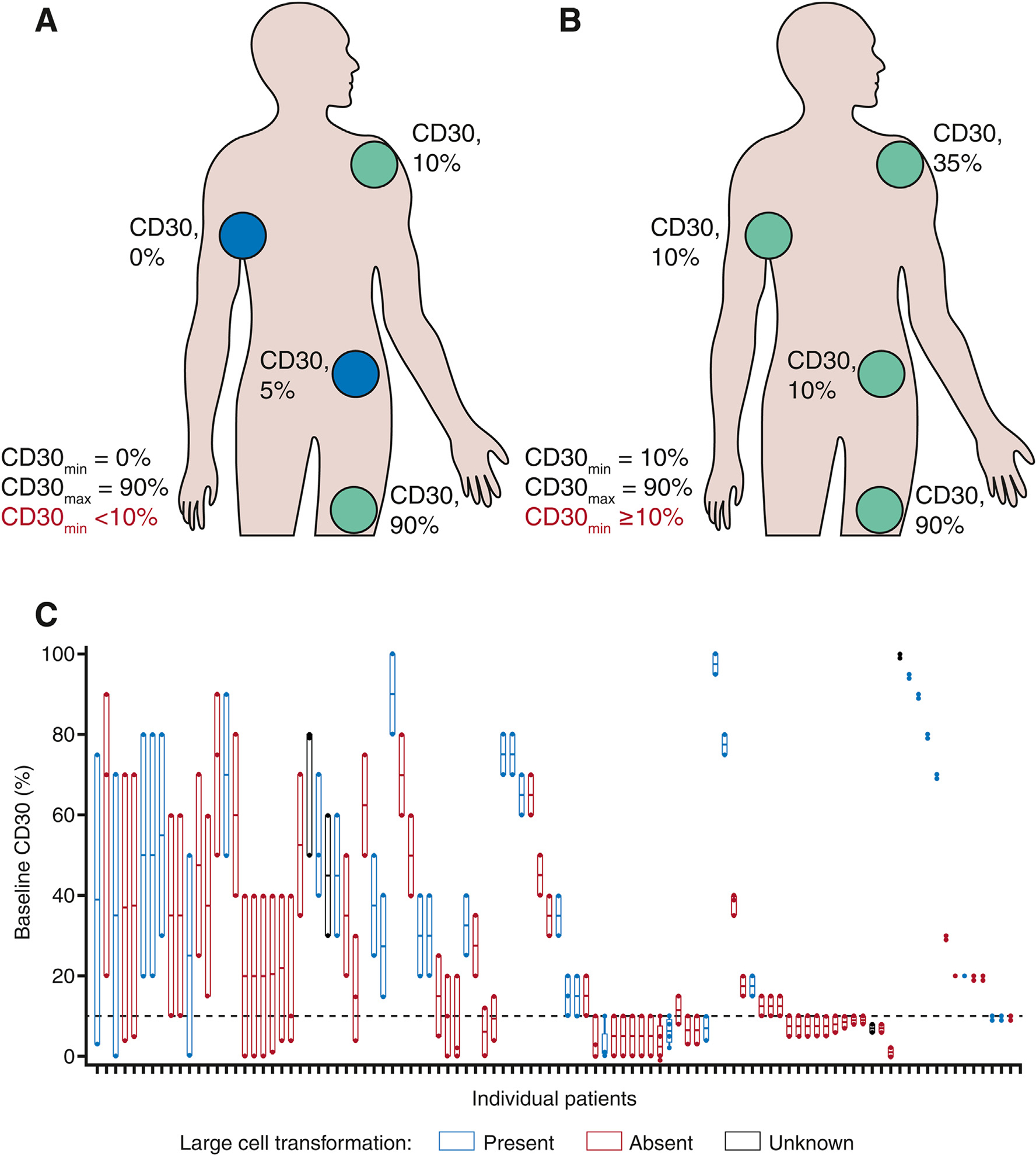
Intra-patient, inter-patient and inter-lesional variability in baseline CD30 expression levels in patients with CD30-positive mycosis fungoides. Patients were allocated to two groups based on their biopsy with the CD30_min_. Patients who had at least one biopsy with <10% CD30 expression (A) were allocated to the CD30_min_ < 10% group, and those with both/all biopsies with ≥10% CD30 expression (B) allocated to CD30_min_ ≥ 10% group. Baseline per patient CD30 expression levels are shown in (C). Each box represents an intra-patient range of CD30 expression for individual patients. Data were plotted from highest to lowest variability in CD30 expression. Horizontal bars within each box represent median CD30 expression among all biopsies tested. The top and bottom of each box represent maximum (CD30_max_) and CD30_min_ values for all biopsies from each patient. The horizontal dashed line at 10% represents the cut-off for enrollment. CD30_max_, maximum CD30 levels; CD30_min_, minimum CD30 levels.

For CD30 assessment, patients with MF required two skin biopsies from separate lesions for eligibility, and additional biopsies were permitted at the investigator’s discretion. Eligibility required only one biopsy to be CD30-positive, defined as ≥ 10% of malignant cells or total lymphoid infiltrate demonstrating membrane, cytoplasmic, and/or Golgi staining pattern for CD30 at any intensity above background staining. Percent positivity was determined based on neoplastic cell staining first. If neoplastic cells could not be easily distinguished from non-neoplastic, then percent positivity was determined based on total lymphocyte staining. CD30 expression levels were assessed by Marise McNeeley (Central Pathology review) utilising the Ventana BerH2 assay. For descriptive purposes, patients’ baseline minimum and average CD30 expression results (CD30_min_ and CD30_avg_) from their skin biopsies are reported. CD30_min_ was derived by taking the average of the result of the biopsy with the lowest CD30 expression from each patient, and the CD30_ave_ was calculated as the average CD30 expression for all biopsies from an individual patient. For the purposes of efficacy analyses, patients were categorised into one of two groups based on the lowest level of CD30 expression (CD30_min_): CD30_min_ < 10% and CD30_min_ ≥ 10% (Fig. 1). Patients categorised as CD30_min_ < 10% had at least one biopsy with CD30 expression below 10% and at least one other biopsy with at least 10% CD30 expression, the threshold for eligibility (Fig. 1A). Patients categorised as CD30_min_ ≥ 10% had ≥10% CD30 expression in both or all biopsies (Fig. 1B).

LCT status at study entry was retrospectively assessed using ≥2 biopsies obtained at the screening. Patients were deemed to have LCT if any single biopsy showed the presence of large cells with nuclei ≥4 times larger than those of normal lymphocytes present in >25% of total dermal infiltrate. LCT status was assessed via central pathologist review (Marise McNeeley), and the LCT-assessment methodology was developed in collaboration with Alejandro Gru (University of Virginia).

Pathologists who provided a central review of CD30 expression and LCT status were blinded to patients’ treatment assignment and clinical outcome.

Treatment-emergent adverse events (AEs) were assessed according to National Cancer Institute Common Terminology Criteria for Adverse Events (CTCAE), version 4.03. Serious AEs were untoward medical occurrences that resulted in death, were lifethreatening, required hospitalisation or prolongation of existing hospitalisation, resulted in persistent or significant disability or capacity, a congenital anomaly/birth defect or an important medical event.

Statistical methods are described in the supplementary file.

## 3. Results

### 3.1. Patients and disposition

Baseline demographics and disease characteristics for the intention to treat population and patient dispositions have been previously reported [14]. Of the 131 patients randomised, 100 had CD30-positive MF (n = 50 in each arm). After randomisation, biopsies from the 100 patients with MF were reassessed for CD30 expression levels using the Ventana IUO assay; baseline biopsy CD30 expression ranged from 0.0% (undetectable) to 100.0%. Three patients (two in the brentuximab vedotin arm and one in the PC arm) had biopsies that were not confirmed as CD30-positive. All three received study treatment, and consequently, they were included in safety analyses but were excluded from the ITT analyses reported in the primary publication. The median CD30_min_ was 10.0% (range: 0.0–100.0%). CD30 levels exhibited high inter-patient and intra-patient variability with several patients exhibiting >60% difference in CD30 expression between biopsies (range: 0–72%) (Fig. 1C). When patients were categorised per baseline, CD30 expression level (CD30_min_ < 10% and CD30_min_ ≥ 10%), 43 patients (43.0%; 22 in the brentuximab vedotin arm and 21 in the PC arm) had ≥1 biopsy with <10% CD30 expression (CD30_min_ < 10%) and 57 patients (57.0%; 28 in the brentuximab vedotin arm and 29 in the PC arm) had all biopsies with ≥10% CD30 expression (CD30_min_ ≥ 10%).

Of the 100 patients with CD30-positive MF, 96 were evaluated for LCT status (n = 48 in each arm) and were included in the response-by-LCT analyses, 4 patients had biopsies that could not be assessed due to crushing artefacts and were therefore classified as having unknown LCT status. Baseline characteristics were well balanced between subgroups (Table 1). In both arms, patients with LCT had a wide range of baseline CD30 levels per patient (Table S1). In general, patients with LCT had higher median value of average CD30 (CD30_avg_) positivity (brentuximab vedotin: 50%; PC: 35%) compared with those without LCT (brentuximab vedotin: 15%; PC: 15%) (Table 1).

### 3.2. Efficacy

#### 3.2.1. Efficacy of brentuximab vedotin by CD30 expression level

**Fig. 2.**
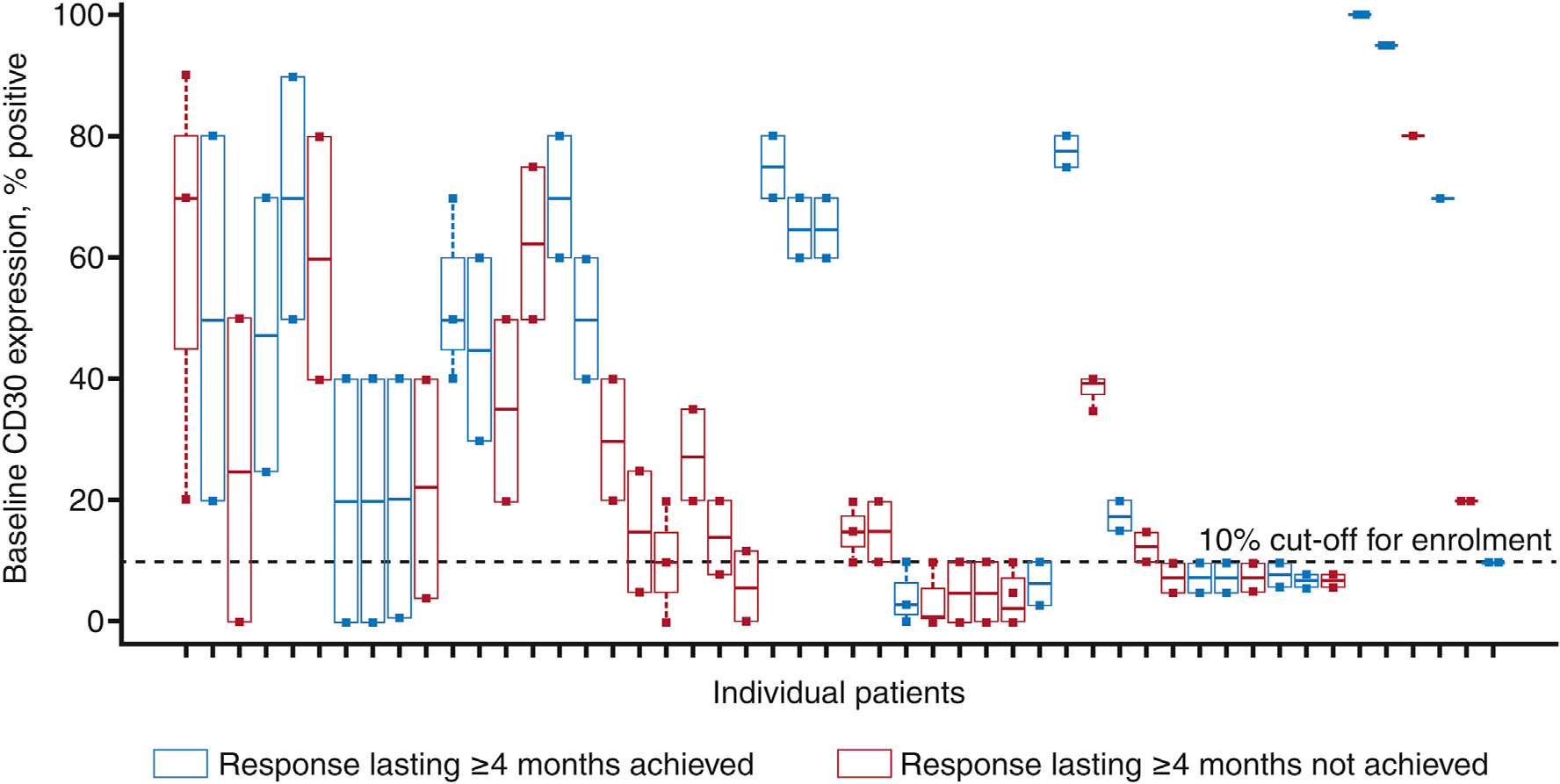
Overall response rate lasting ≥4 months in patients with CD30-positive mycosis fungoides treated with brentuximab vedotin. Per patient objective response rate lasting ≥4 months and minimum baseline (CD30_min_) levels. Each box represents an intra-patient range of CD30 expression for individual patients; individual dots represent CD30 expression from individual biopsies at baseline. Data were plotted from highest to lowest variability in CD30 expression. Horizontal bars within each box represent median CD30 expression among all biopsies tested. The top and bottom of each box represent 75th and 25th percentiles; upper and lower ends of vertical dashed lines represent maximum and minimum values (for patients with two biopsies 75th and 25th percentiles overlapped maximum and minimum values). The horizontal dashed line at 10% represents the cut-off for enrollment.

Recognising the high inter-patient and intra-patient variability of CD30 expression levels at baseline, the relationship between baseline CD30 levels and ORR4 was assessed on a per-patient basis. Among the 50 patients with CD30-positive MF treated with brentuximab vedotin, 25 patients (50.0%) achieved ORR4 criteria independent of baseline CD30 expression levels (Fig. 2). Brentuximab vedotin was superior to PC in the CD30_min_ < 10% subgroup (ORR4 40.9% versus 9.5%; Δ31.4% [95% CI 2.8–58.1]) and CD30_min_ ≥ 10% subgroup (ORR4 57.1% versus 10.3%; Δ46.8% [95% CI: 20.6–67.0]) (Table 2).

**Fig. 3.**
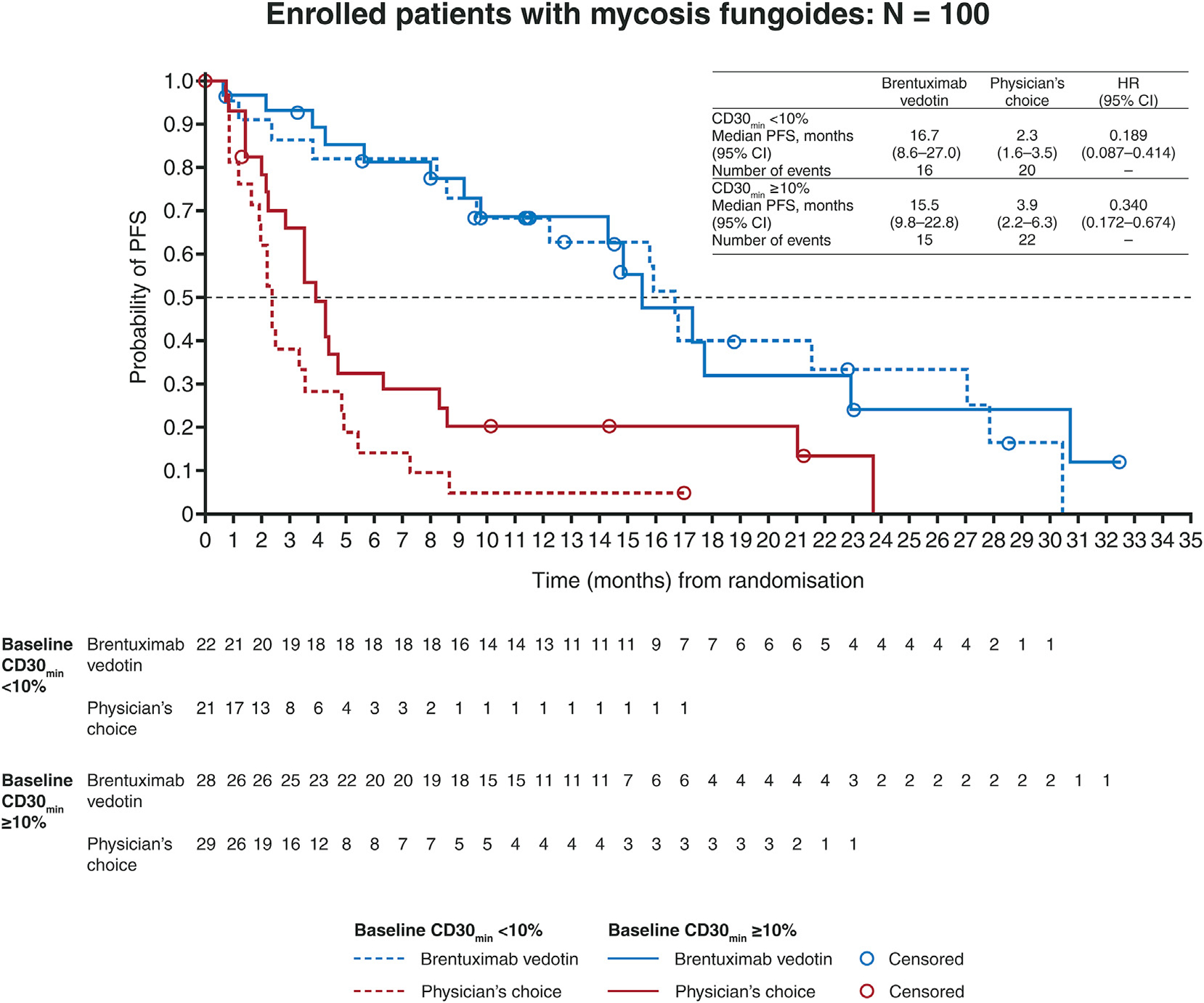
Comparison of PFS with brentuximab vedotin versus physician’s choice by baseline CD30 expression level in patients with CD30-positive mycosis fungoides. CD30min, minimum CD30; CI, confidence interval; HR, hazard ratio; PFS, progression-free survival.

Median PFS in the brentuximab vedotin arm was higher than that in the PC arm, regardless of baseline CD30 expression. For patients with CD30_min_ < 10% median PFS with brentuximab vedotin was 16.7 months (95% CI: 8.6–27.0) versus 2.3 months (95% CI: 1.6–3.5) with PC (HR: 0.189; 95% CI: 0.087–0.414). For patients with CD30_min_ ≥ 10%, median PFS was 15.5 months with brentuximab vedotin (95% CI: 9.8–22.8) versus 3.9 months with PC (95% CI: 2.2–6.3) with HR: 0.340 (95% CI: 0.172–0.674) (Fig. 3). The CD30_min_ and CD30_max_ levels at baseline had no discernible effect on whether patients achieved an ORR4 or not (Fig. S1).

#### 3.2.2. Efficacy of brentuximab vedotin by LCT status

ORR4 was consistently higher with brentuximab vedotin versus PC in patients with LCT (n = 11 [64.7%] versus n = 3 [17.6%]) and those without LCT (n = 12 [38.7%] versus n = 2 [6.5%]) (Table 2). Within the brentuximab vedotin arm, a higher proportion of patients with LCT achieved an ORR4 than those without LCT (64.7% [n = 11] versus 38.7% [n = 12]) (Table S1), although the difference was not significant (p = 0.155). Median PFS was improved with brentuximab vedotin versus PC in patients with LCT (15.5 months [95% CI: 9.1–22.8] versus 2.8 months [95% CI: 1.4–7.3]; p = 0.002) and without LCT (16.1 months [95% CI: 8.6–21.6] versus 3.5 months [95% CI: 2.2–4.3]; p < 0.001) (Table 2).

Among patients with LCT, the median CD30_avg_ expression was 65% in patients who achieved ORR4 versus 20% in those who did not (Table S1). Of the patients with LCT who achieved ORR4, 9/11 patients in the brentuximab vedotin arm had CD30_avg_ ≥ 40.0%, but responses were also noted in the 2 patients with low CD30_avg_ (10.0% and 17.5%) (Fig. S2A). In the PC arm 3 patients with a range of CD30_avg_ values achieved OR lasting ≥4 months (Fig. S2B).

## 3.3. Safety analyses

In the primary analysis of the ALCANZA safety population, any grade adverse events (AEs) occurred in 95% of 66 patients in the brentuximab vedotin arm and 90% of the 62 patients in the PC arm; grade 3–4 AE rates were 41% and 47% in the brentuximab vedotin and PC arms, respectively [14]. In the brentuximab vedotin arm, peripheral neuropathy was the most frequent any grade AE, occurring in 67% of patients in the brentuximab vedotin group versus 6% in the PC arm. In the PC arm, the AE profiles were different between patients treated with methotrexate and bexarotene. The most frequent any grade AE in methotrexate-treated patients was pyrexia (28% [4% grade 3]), whereas the most frequent AE in bexarotene-treated patients was hypertriglyceridaemia (30% [14% grade 3, 8% grade 4]) [14].

Table 3 presents a summary of treatment-emergent AEs occurring in patients with MF categorised by CD30 expression levels per the current analysis. Overall, AE incidences were similar in the brentuximab vedotin and PC treatment arms regardless of CD30 expression levels. Peripheral neuropathy occurred more often in brentuximab vedotin-treated patients with similar rates between CD30_min_ < 10% and CD30_min_ ≥ 10% (68.2% and 67.9%, respectively). Rates of grade ≥3 AEs were not significantly different in patients with CD30_min_ < 10% compared with those with CD30_min_ ≥ 10% in the brentuximab vedotin arm (50.0% versus 35.7%; p = 0.4670) and the PC arm (57.1% versus 32.1%; p = 0.1447). The incidence of serious AEs exhibited a similar pattern with numerically higher incidences in patients with CD30_min_ <10% compared with those with CD30_min_ ≥ 10% in the brentuximab vedotin arm (31.8% versus 28.6%) and the PC arm (42.9% versus 17.9%). There was no difference in safety with respect to LCT status.

## 4. Discussion

Previous studies have demonstrated responses to brentuximab vedotin in patients with MF across a range of CD30 expression levels, including 0% [7,8]. The current analyses found that despite high inter-patient and intra-patient variability in baseline CD30 expression levels of patients with MF, a higher proportion of patients treated with brentuximab vedotin patients achieved ORR4 compared with those who received PC, and median PFS values were higher with brentuximab vedotin, regardless of baseline CD30 expression levels as assessed by CD30_min_. Clinical responses lasting at least 4 months (ORR4 criteria) were observed across all CD30 expression levels. In addition, AE profiles were generally comparable, irrespective of baseline CD30 expression levels.

The results observed in the ALCANZA study were consistent with previously reported investigator-initiated studies [6–8] where significant clinical activity was observed in patients with low-levels (<10%) of skin CD30 expression. In ALCANZA, demonstration of the effectiveness of brentuximab vedotin in patients who have low (<10%) or visually undetectable levels of CD30 by IHC in one biopsy may be due to lack of sensitivity of the assay used to detect cell-surface CD30 expression. In another CTCL study, the use of a more sensitive detection methodology (e.g. multispectral imaging) suggests that appreciable CD30 expression may be present in up to 95% of IHC-negative biopsies [8]. A post-marketing commitment for the approval of brentuximab vedotin + cyclophosphamide, doxorubicin, and prednisolone (CHP) in front-line sALCL or other CD30-expressing peripheral T-cell lymphomas is to develop a clinically validated *in vitro* diagnostic for CD30 expression to inform patient selection. In the meantime, standard IHC detection remains an appropriate tool for characterising CD30-expressing malignancies, though guidelines may be helpful.

In ALCANZA, the presence of highly variable CD30 expression between different lesions within the same patient (intra-patient variability) may also contribute to why patients identified as “CD30-negative” in a single biopsy may benefit from brentuximab vedotin. With 43% of MF patients enrolled having at least one baseline biopsy with <10% CD30 expression, multiple biopsies may be considered for testing; however, assessment of CD30 expression levels in an investigator-initiated trial utilising multiple skin biopsies demonstrated similar intra-patient variability in the CD30 expression levels [6,8]. Other studies have postulated that alternative, CD30-independent tumour killing mechanisms may contribute to the antitumour activity of brentuximab vedotin. These include antibody-dependent cellular phagocytosis, immunogenic cell death, the bystander effect and depletion of CD30-positive T regulatory cells [15–20]. In the ALCANZA study, there does not appear to be a level of CD30 expression that is predictive of response to brentuximab vedotin for patients with MF making the determination of a threshold level uncertain. Interpretation of these findings may be limited as the ALCANZA study excluded patients with <10% CD30 expression per central review, and patients may have been selected for screening based upon the local evaluation of CD30 expression.

LCT in patients with MF is largely seen as an independent prognostic factor for a less favourable outcome in patients with MF, being associated with aggressive disease and inferior prognosis [10–13,21]. Contrary to this, the current sub-analysis of patients with MF in the ALCANZA study found that the superior efficacy of brentuximab vedotin compared with PC was largely unaffected by the presence or absence of LCT. In terms of the ALCANZA primary endpoint, ORR4, patients with MF and baseline LCT achieved higher ORR4 than those without LCT in both the brentuximab vedotin and the PC arms. Within each arm, median PFS was comparable between LCT subgroups, suggesting no clinically meaningful impact of LCT status on PFS. Interpretation of results per LCT status may, however, be limited by low sample size and intra-patient heterogeneity of detectable LCT in individual biopsies. In other words, patients without LCT may actually have false-negative biopsies based on selection bias relating to the biopsy site. Within each arm, median PFS was comparable between LCT subgroups, suggesting no clinically meaningful impact of LCT status on PFS.

Finally, the safety profiles of brentuximab vedotin and PC in patients with MF were similar and largely unaffected by baseline CD30 status; rates of serious AEs were similar between the CD30_min_ ≥ 10% and CD30_min_ < 10% subgroups. Peripheral neuropathy is a known effect of brentuximab vedotin treatment and is generally reversible [14]. There was no meaningful difference in rates of peripheral neuropathy in each of the CD30 subgroups evaluated.

In conclusion, these results indicate that in the ALCANZA study population, CD30 expression is present in most of the patients with MF, both with and without LCT. Given that treatment responses were observed across the entire range of CD30 expression, study outcomes demonstrate a consistently favourable benefit/risk profile for brentuximab vedotin in patients, irrespective of baseline CD30 expression levels and LCT status.

## Supplementary Material

1

2

## Figures and Tables

**Table 1:** Baseline characteristics of patients with LCT-evaluable CD30-positive mycosis fungoides.

	Brentuximab vedotin (n = 48)	Physician’s choice (n = 48)

Male, n (%)	27 (56)	26 (54)
Median age, years (range)	56 (22–83)	59 (22–81)
LCT present, n (%)	17 (35)	17 (35)
LCT absent, n (%)	31 (65)	31 (65)
Overall staging, n (%)		
IA–IIA	15 (31)	19 (40)
IIB	19 (40)	18 (38)
III	4 (8)	2 (4)
IV	9 (19)	9 (19)
Unknown	1 (2)	–

LCT present	(n = 17)	(n = 17)

Median CD30_avg_,% (range)	50.00 (3.0–95.0)	35.00 (6.3–97.5)
Median CD30_min_,% (range)	30.00 (0.0–95.0)	20.00 (0.0–95.0)

LCT absent	(n = 31)	(n = 31)

Median CD30_avg_,% (range)	15.00 (3.8–70.0)	15.00 (1.0–71.7)
Median CD30_min_,% (range)	5.00 (0.0–60.0)	8.00 (0.0–50.0)

CD30_avg_, CD30 average levels; CD30_min_, minimum CD30 levels; LCT, large cell transformation.

**Table 2:** Efficacy of brentuximab vedotin and PC by CD30 expression and LCT status.

Treatment	CD30_min_ < 10% (n = 43)		CD30_min_ ≥ 10% (n = 57)	
	
	Brentuximab vedotin (n = 22)	Physician’s choice (n = 21)	Brentuximab vedotin (n = 28)	Physician’s choice (n = 29)

ORR4, n (%)	9 (40.9)	2 (9.5)	16 (57.1)	3 (10.3)
Δ versus PC, % (95% CI)	31.4 (2.8–58.1)		46.8 (20.6–67.0)	
Median PFS, months (95% CI)	16.7 (8.6–27.0)	2.3 (1.6–3.5)	15.5 (9.8–22.8)	3.9 (2.2–6.3)
HR (95% CI)	0.189 (0.087–0.414)		0.340 (0.172–0.674)	

Treatment	LCT present (n = 34)		LCT absent (n = 62)	
	
	Brentuximab vedotin (n = 17)	Physician’s choice (n = 17)	Brentuximab vedotin (n = 31)	Physician’s choice (n = 31)

ORR4 per IRF, n (%)	11 (64.7)	3 (17.6)	12 (38.7)	2 (6.5)
Median PFS, months (95% CI)	15.5 (9.1–22.8)	2.8 (1.4–7.3)	16.1 (8.6–21.6)	3.5 (2.2–4.3)
Median CD30_min_, % (range)	30.0 (0–95.0)	20.0 (0–95.0)	5.0 (0–60.0)	8.0 (0–50.0)

CD30_min_, minimum CD30 levels; CI, confidence interval; HR, hazard ratio; IRF, independent review facility; LCT, large cell transformation; ORR4, objective response rate lasting ≥4 months; PC, physician’s choice; PFS, progression-free survival.

**Table 3:** Overall summary of treatment-emergent AEs by CD30 expression level.

Treatment	Brentuximab vedotin (n = 50)		Physician’s choice (n = 49)	
CD30_min_ subgroup	CD30_min_ < 10% (n = 22)	CD30_min_ ≥ 10% (n = 28)	CD30_min_ < 10% (n = 21)	CD30_min_ ≥ 10% (n = 28)

Any treatment-emergent AE, n (%)	22 (100.0)	28 (100.0)	20 (95.2)	23 (82.1)
Grade ≥3 AE, n (%)	11 (50.0)	10 (35.7)	12 (57.1)	9 (32.1)
Serious AE, n (%)	7 (31.8)	8 (28.6)	9/(42.9)	5 (17.9)
Peripheral neuropathy, n (%)	15 (68.2)	19 (67.9)	0	2 (7.1)

AE, adverse event; CD30_min_, minimum CD30 levels.

## Data Availability

The datasets, including the redacted study protocol, redacted statistical analysis plan and individual participants data supporting the results reported in this article, will be made available within three months from the initial request to researchers who provide a methodologically sound proposal. The data will be provided after its de-identification in compliance with applicable privacy laws, data protection and requirements for consent and anonymisation.
