# Supplementary material for: Response to brentuximab vedotin versus physician’s choice by CD30 expression and large cell transformation status in patients with mycosis fungoides: An ALCANZA sub-analysis
Source: Eur J Cancer. Author manuscript; Available in PMC 2022 Aug 3. (PMC9347228; doi:10.1016/j.ejca.2021.01.054)
Supplement: 1 [file NIHMS1819586-supplement-1.docx]

**Supplementary File**

**Article title:** Response to brentuximab vedotin versus physician’s choice by CD30 expression and large cell transformation status in patients with mycosis fungoides: an ALCANZA sub-analysis

**Supplementary methods**

1. **CD30 assessment**

Patients with mycosis fungoides (MF) required two skin biopsies from separate lesions for eligibility, additional biopsies were permitted at the investigator’s discretion. Eligibility required only one biopsy to be CD30-positive, defined as ≥10% of malignant cells or total lymphoid infiltrate demonstrating membrane, cytoplasmic and/or Golgi staining pattern for CD30 at any intensity above background staining. Percent positivity was determined based on neoplastic cell staining first. If neoplastic cells could not be easily distinguished from non-neoplastic, then percent positivity was determined based on total lymphocyte staining. CD30 expression levels were assessed by Marise McNeeley (Central Pathology review) utilising the Ventana BerH2 assay. For descriptive purposes, patients’ baseline minimum and average CD30 expression results (CD30_min_ and CD30_avg_) from their skin biopsies are reported. CD30_min_ was derived by taking the average of the result of the biopsy with the lowest CD30 expression from each patient, the CD30_avg_ was calculated as the average CD30 expression for all biopsies from an individual patient.

1. **Statistical methods**

In order to evaluate efficacy of brentuximab vedotin at lower levels of CD30 expression, the per-patient skin biopsy with the lowest level of CD30 expression was used to dichotomise MF patients into two groups: CD30_min_ <10% versus CD30_min_ ≥10%. Overall responses lasting ≥4 months and progression-free survival were assessed as described in the primary ALCANZA publication [1], in patients with MF by CD30_min_ <10% versus CD30_min_ ≥10% in the brentuximab vedotin and physician’s choice arms. All analyses were descriptive only. Safety analyses were performed for all patients with MF who received at least one dose of study drug and are presented by CD30_min_ <10% versus CD30_min_ ≥10% in both arms.

**Supplementary figures**


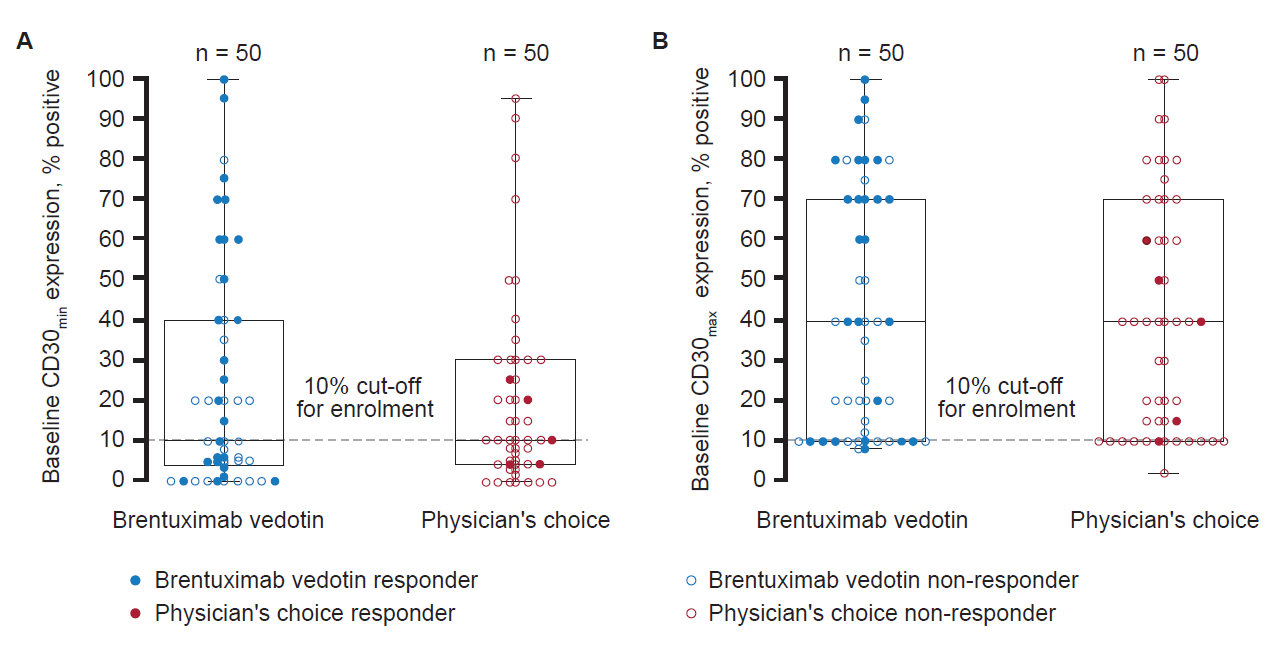
 Fig. S1. ORR4 in response to brentuximab vedotin treatment by baseline CD30_min_ (A) and CD30_max_ (B). Top and bottom of each box represent the 75th and 25th percentiles; bands within each box represent the median; upper and lower bars represent the maximum and minimum values. Responding patients (closed circles) were those achieving ORR4 (non-responding patients [open circles] did not). Three patients enrolled based on results of the Quest Diagnostics clinical trial assay, but who had CD30_max_ <10% upon retesting with the Ventana Investigational Use Only test, are evident in panel (B). CD30_max_, maximum CD30 levels; CD30_min_, minimum CD30 levels; ORR4, overall responses lasting ≥4 months.


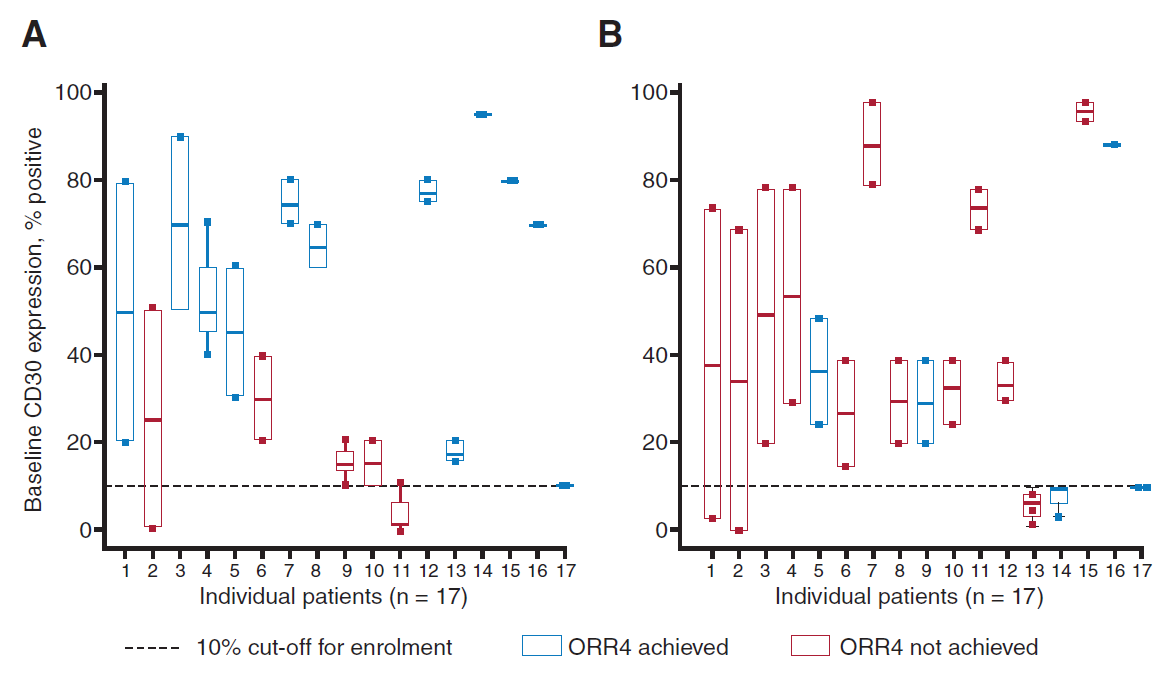
 Fig. S2. Proportion of patients with CD30-positive mycosis fungoides and LCT at baseline who achieved an ORR4 with brentuximab vedotin and physician’s choice. (A) Patients with LCT at baseline who received brentuximab vedotin. (B) Patients with LCT at baseline who received physician’s choice. LCT, large cell transformation; ORR4, overall responses lasting ≥4 months.

**Supplementary tables**

Table S1

LCT, CD30 expression, best response, ORR4 and duration of response in patients from the brentuximab vedotin arm.

| LCT: present (n = 17) | | | | |
| --- | --- | --- | --- | --- |
| Average CD30 expression per patient, % (range) | Best response | ORR4 achieved per IRF | DOR, months | PFS, months |
| 3 (0–10) | SD | No |  | 9.59 (C) |
| 10 (10–10) | PR | Yes | 20.60 (C) | 22.83 (C) |
| 15 (10–20) |  | No |  | 36.01 |
| 15 (10–20) | PD | No |  | 2.14 (C) |
| 17.5 (15–20) | PR | Yes | 9.53 | 11.63 |
| 25 (0–50) | SD | No |  | 8.18 (C) |
| 30 (20–40) |  | No |  | 14.26 (C) |
| 45 (30–60) | PR | Yes | 22.14 | 23.49 (C) |
| 50 (20–80) | CR | Yes | 29.34 (C) | 30.72 (C) |
| 53.33 (40–70) | PR | Yes | 8.54 (C) | 14.88 (C) |
| 65 (60–70) | PR | Yes | 7.29 (C) | 7.98 (C) |
| 70 (50–90) | PR | Yes | 7.06 (C) | 9.13 (C) |
| 70 (70–70) | PR | Yes | 4.8 | 5.65 |
| 75 (70–80) | PR | Yes | 10.84 | 16.46 (C) |
| 77.5 (75–80) | PR | Yes | 15.57 (C) | 17.64 (C) |
| 80 (80–80) | SD | No |  | 9.92 |
| 95 (95–95) | CR | Yes | 13.44 (C) | 15.51 (C) |
| LCT: absent (n = 31) | | | | |
| Average CD30 expression per patient, % (range) | Best response | ORR4 achieved per IRF | DOR, months | PFS, months |
| 3.75 (0–10) | PD | No |  | 27.86 (C) |
| 4.33 (0–10) | CR | Yes | 21.82 | 22.93 |
| 5 (0–10) | SD | No |  | 2.33 (C) |
| 5 (0–10) | SD | No |  | 27.04 (C) |
| 6 (0–2) | PD | No |  | 0.72 (C) |
| 6.5 (3–10) | PR | Yes | 9.69 (C) | 15.90 (C) |
| 7 (6–8) |  | No |  | 12.22 (C) |
| 7.5 (5–10) | PD | No |  | 1.18 (C) |
| 7.5 (5–10) | SD | No |  | 30.42 (C) |
| 7.5 (5–10) | PR | Yes | 14.36 (C) | 16.66 (C) |
| 7.5 (5–10) | PR | Yes | 18.79 (C) | 21.55 (C) |
| 8 (6–10) | PR | Yes | 11.53 (C) | 15.77 (C) |
| 10 (0–20) | PR | No | 1.64 (C) | 3.84 (C) |
| 12.5 (10–15) | PR | No | 1.51 (C) | 3.75 (C) |
| 14 (8–20) | SD | No |  | 12.06 |
| 15 (5–25) | PR | No | 3.25 | 16.82 (C) |
| 20 (20–20) |  | No |  | 3.42 |
| 20 (0–40) | PR | Yes | 9.79 (C) | 16.10 (C) |
| 20 (0–40) | CR | Yes | 16.85 | 18.96 |
| 20.5 (1–40) | PR | Yes | 10.94 | 15.90 |
| 22 (4–40) | PR | No | 4.21 (C) | 8.57 (C) |
| 27.5 (20–35) |  | No |  | 0.56 (C) |
| 35 (20–50) | SD | No |  | 0.82 |
| 38.33 (35–40) | PR | No | 0.69 (C) | 4.24 (C) |
| 47.5 (25–70) | PR | Yes | 15.08 (C) | 17.25 (C) |
| 50 (40–60) | PR | Yes | 5.32 | 11.56 |
| 60 (40–80) | SD | No |  | 26.38 |
| 60 (20–90) | PR | No | 5.55 (C) | 9.82 (C) |
| 62.5 (50–75) | PR | No | 3.48 (C) | 5.65 (C) |
| 65 (60–70) | PR | Yes | 11.89 (C) | 15.44 (C) |
| 70 (60–80) | CR | Yes | 32.13 | 33.84 |

C, censored; CR, complete response; DOR, duration of response; IRF, independent review facility; LCT, large cell transformation; ORR4, objective response lasting ≥4 months; PD, progressive disease; PFS, progression-free survival; PR partial response; SD, stable disease.

**References**

[1] Prince HM, Kim YH, Horwitz SM, Dummer R, Scarisbrick J, Quaglino P, et al. Brentuximab vedotin or physician's choice in CD30-positive cutaneous T-cell lymphoma (ALCANZA): an international, open-label, randomised, phase 3, multicentre trial. The Lancet 2017;390:555–66. <https://doi.org/10.1016/S0140-6736(17)31266-7>.
