# Supplementary figures and images for: Response to brentuximab vedotin versus physician’s choice by CD30 expression and large cell transformation status in patients with mycosis fungoides: An ALCANZA sub-analysis
Source: Eur J Cancer. Author manuscript; Available in PMC 2022 Aug 3. (PMC9347228; doi:10.1016/j.ejca.2021.01.054)

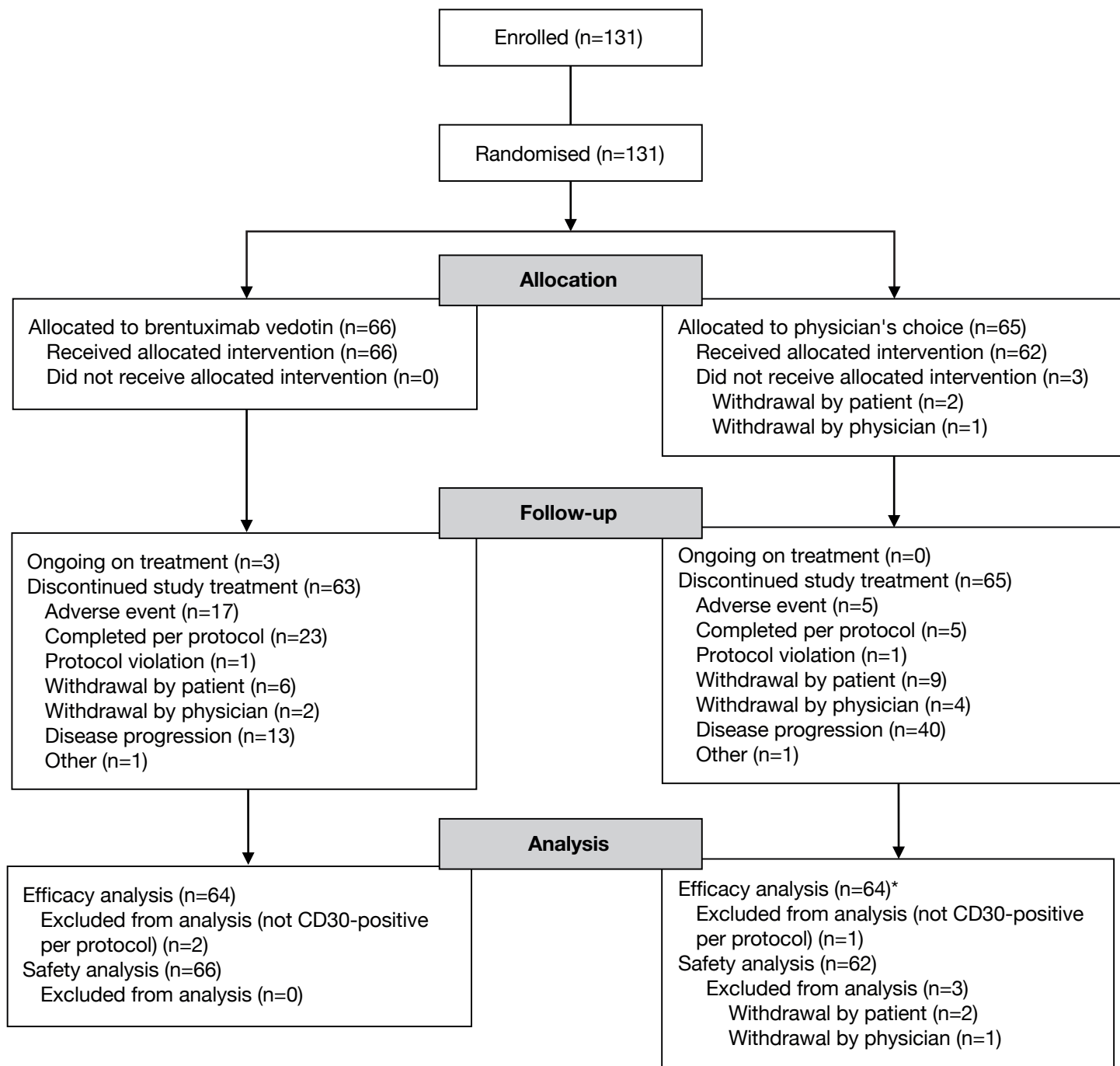

Supplement: 2 [file NIHMS1819586-supplement-2.pdf]
